# Lanthanide-Dependent Regulation of Methylotrophy in *Methylobacterium* *aquaticum* Strain 22A

**DOI:** 10.1128/mSphere.00462-17

**Published:** 2018-01-24

**Authors:** Sachiko Masuda, Yutaka Suzuki, Yoshiko Fujitani, Ryoji Mitsui, Tomoyuki Nakagawa, Masaki Shintani, Akio Tani

**Affiliations:** a Institute of Plant Science and Resources, Okayama University, Okayama, Japan; b Advanced Low Carbon Technology Research and Development Program, Japan Science and Technology Agency, Tokyo, Japan; c Department of Computational Biology and Medical Sciences, University of Tokyo, Chiba, Japan; d Department of Biochemistry, Faculty of Science, Okayama University of Science, Okayama, Japan; e Faculty of Applied Biological Science, Gifu University, Gifu, Japan; f Department of Engineering, Graduate School of Integrated Science and Technology, Shizuoka University, Shizuoka, Japan; Martin Luther University of Halle-Wittenberg

**Keywords:** lanthanide, methanol dehydrogenase, methylotrophs, *xoxF*

## Abstract

*Methylobacterium* species are representative of methylotrophic bacteria. Their genomes usually encode two types of methanol dehydrogenases (MDHs): MxaF and XoxF. The former is a Ca^2+^-dependent enzyme, and the latter was recently determined to be a lanthanide-dependent enzyme that is necessary for the expression of *mxaF*. This finding revealed the unexpected and important roles of lanthanides in bacterial methylotrophy. In this study, we performed transcriptome sequencing (RNA-seq) analysis using *M. aquaticum* strain 22A grown in the presence of different lanthanides. Expression of *mxaF* and *xoxF1* genes showed a clear inverse correlation in response to La^3+^. We observed downregulation of formaldehyde oxidation pathways, high formaldehyde dehydrogenase activity, and low accumulation of formaldehyde in the reaction with cells grown in the presence of La^3+^; this might be due to the direct oxidation of methanol to formate by XoxF1. Lanthanides induced the transcription of AT-rich genes, the function of most of which was unknown, and genes possibly related to cellular survival, as well as other MDH homologues. These results revealed not only the metabolic response toward altered primary methanol oxidation, but also the possible targets to be investigated further in order to better understand methylotrophy in the presence of lanthanides.

**IMPORTANCE** Lanthanides have been considered unimportant for biological processes. In methylotrophic bacteria, however, a methanol dehydrogenase (MDH) encoded by *xoxF* was recently found to be lanthanide dependent, while the classic-type *mxaFI* is calcium dependent. XoxF-type MDHs are more widespread in diverse bacterial genera, suggesting their importance for methylotrophy. *Methylobacterium* species, representative methylotrophic and predominating alphaproteobacteria in the phyllosphere, contain both types and regulate their expression depending on the availability of lanthanides. RNA-seq analysis showed that the regulation takes place not only for MDH genes but also the subsequent formaldehyde oxidation pathways and respiratory chain, which might be due to the direct oxidation of methanol to formate by XoxF. In addition, a considerable number of genes of unknown function, including AT-rich genes, were found to be upregulated in the presence of lanthanides. This study provides first insights into the specific reaction of methylotrophic bacteria to the presence of lanthanides, emphasizing the biological relevance of this trace metal.

## Abstract (precis)

Lanthanides have been considered unimportant for biological processes. In methylotrophic bacteria, however, a methanol dehydrogenase (MDH) encoded by *xoxF* was recently found to be lanthanide dependent, while the classic-type *mxaFI* is calcium dependent. XoxF-type MDHs are more widespread in diverse bacterial genera, suggesting their importance for methylotrophy. *Methylobacterium* species, representative methylotrophic and predominating alphaproteobacteria in the phyllosphere, contain both types and regulate their expression depending on the availability of lanthanides. RNA-seq analysis showed that the regulation takes place not only for MDH genes but also the subsequent formaldehyde oxidation pathways and respiratory chain, which might be due to the direct oxidation of methanol to formate by XoxF. In addition, a considerable number of genes of unknown function, including AT-rich genes, were found to be upregulated in the presence of lanthanides. This study provides first insights into the specific reaction of methylotrophic bacteria to the presence of lanthanides, emphasizing the biological relevance of this trace metal.

## INTRODUCTION

*Methylobacterium* species are facultative methylotrophic alphaproteobacteria. They are ubiquitous in the natural environment, being found in soil, dust, water, air, and plants (1, 2). The metabolic pathway of methanol in *Methylobacterium* species has long been studied using *Methylobacterium extorquens* strain AM1 as a model (3). This strain, like all Gram-negative methylotrophs, oxidizes methanol to formaldehyde with pyrroloquinoline quinone (PQQ)-dependent methanol dehydrogenases (MDHs). Formaldehyde is further oxidized to formate and to CO_2_. In the case of *Methylobacterium* species (and many methylotrophic alphaproteobacteria) (4), formate is converted into methylene-H_4_F and fixed in the serine cycle for biosynthesis of cell constituents. *Methylobacterium* species usually contain two types of MDHs, encoded by *mxaFI* and *xoxF*. MxaFI consists of large (MxaF) and small (MxaI) subunits in a Ca^2+^-dependent PQQ-containing MDH (5). MxaFI has been considered the main and indispensable MDH under laboratory conditions. Although the exact function of XoxF was previously a mystery (6, 7), recent findings have revealed that it is a lanthanide-dependent MDH (8–11). XoxF is the first enzyme that has been shown to require lanthanide metals for its catalytic activity (12).

The genome of strain AM1 carries two *xoxF* genes (*xoxF1* and *xoxF2*) sharing 87% amino acid sequence identity. An *xoxF1* mutant showed delayed growth on methanol (6, 7), and an *mxaF* mutant can grow in the presence of lanthanides due to intact *xoxF1* (10). *xoxF1* is induced only by light lanthanides (La^3+^, Ce^3+^, Pr^3+^, and Nd^3+^), at concentrations as low as 50 to 100 nM (13). Although an *xoxF2* mutant showed no growth defect on methanol, an *xoxF1 xoxF2* double mutant did not grow on methanol at all; these genes are required for the expression of *mxaF* (6). Suppression mutants that regained growth on methanol were obtained from the double mutant, possibly due to the loss of an unknown gene function (6). In the case of the methanotrophic *Methylomicrobium buryatense* strain 5GB1C, the causative mutation in similar suppression mutants was identified in *mxaY* encoding a response regulator. This mutation partially controls the expression of MxaB, an orphan response regulator necessary for *mxa* operon expression (14, 15). Thus, the molecular mechanism for the metal-dependent expression of MDHs remains to be studied.

*xoxF* is found in a wider array of bacterial genomes than *mxaF* (16). XoxF proteins can be classified into at least five groups: XoxF1 from NC10 and *Proteobacteria*; XoxF2 from NC10, LW, and *Verrucomicrobia*; XoxF3 from *Proteobacteria*; XoxF4 from *Methylophilaceae*; and XoxF5 from *Proteobacteria* (note that these names are for phylogenetic groups, not specific genes) (4). They all belong to a larger PQQ-dependent alcohol dehydrogenase family, and MxaF comprises only one branch of them. Since lanthanides are not rare in the environment (17–19), methylotrophy in the presence of lanthanides might be closer to their natural lifestyle than that under lanthanide-free laboratory conditions.

Although lanthanide-dependent MDH expression has been studied, the regulation of methylotrophy in the presence of lanthanides and the genome-wide responses to lanthanides in *Methylobacterium* species have not yet been clarified. In this study, we revealed the transcriptome of *M. aquaticum* strain 22A using transcriptome sequencing (RNA-seq) to determine the genomic responses to lanthanides. The strain was isolated from a moss, *Racomitrium japonicum* (20), and capable of promoting the growth of various plants, and its complete genome information is available (21). Phylogenetically, it is distantly related to *M. extorquens* within the genus, and members of the species have been isolated frequently from plants, including rice (22). In addition to lanthanum (La) as a light lanthanide, we also used heavier metals, holmium (Ho) and lutetium (Lu), to examine their effect on the transcriptome. The analysis revealed the lanthanide-dependent regulation of MDHs and methylotrophy systems, as well as the responses of other genes whose functions are largely unknown. They therefore represent targets for investigation in order to better understand life in the presence of lanthanides.

## RESULTS

### Growth characteristics of strain 22A in the presence of lanthanides.

**FIG 1:**
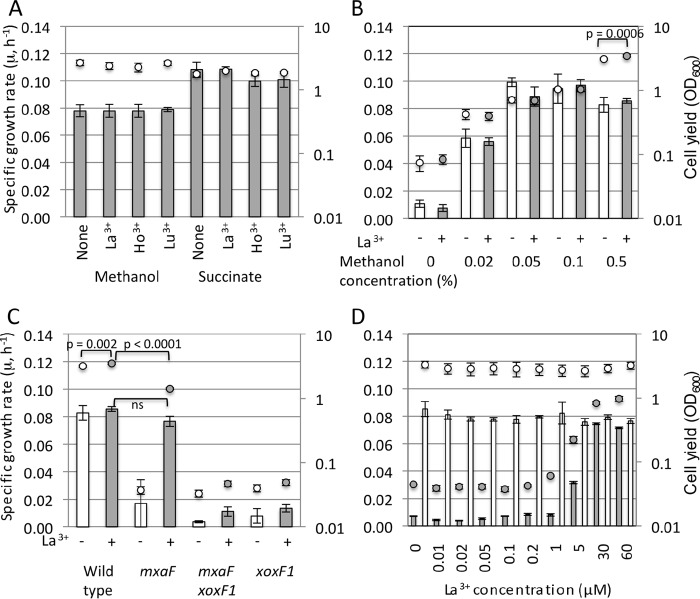
Growth characteristics of strain 22A and its MDH mutant derivatives. (A) Growth of strain 22A on 0.5% methanol or succinate in the presence of 30 µM La^3+^, Ho^3+^, and Lu^3+^. Cell yield is shown as OD_600_ values at 120 h. (B) Growth of strain 22A on methanol at different concentrations in the absence (open symbols) or presence (gray symbols) of 30 µM La^3+^. Cell yield is shown as OD_600_ values at 144 h. Statistical analysis was done using two-way analysis of variance with Bonferroni’s *post hoc* test. Only a *P* value of <0.05 for the effect of La^3+^ addition is shown. (C) Growth of the strain 22A wild type and *mxaF*, *mxaF xoxF1*, and *xoxF1* mutants on 0.5% methanol in the absence (open symbols) or presence (gray symbols) of 30 µM La^3+^. Cell yield is shown as OD_600_ values at 161 h. Statistical analysis was done using two-way analysis of variance with Tukey’s *post hoc* tests. Only *P* values of interest are shown. ns, not significant statistically (*P* > 0.05) between the wild type and the *mxaF* mutant in the presence of La^3+^. (D) Growth of the strain 22A wild type (open symbols) and *mxaF* mutant (gray symbols) on 0.5% methanol in the presence of different concentrations of La^3+^. Cell yield is shown as OD_600_ values at 209 h. All experiments were performed in triplicate, and error bars show the standard deviation (SD). The specific growth rate and cell yield are shown as bars and circles, respectively.

We observed the growth of strain 22A on 0.5% methanol or 0.5% succinate in the presence of 30 µM lanthanides (Fig. 1A). The specific growth rate was not affected by the presence of lanthanides, irrespective of the carbon sources. As shown below, only La^3+^ among the tested lanthanides affected methylotrophy gene expression; therefore, the growth characteristics were investigated only with La^3+^. The specific growth rate and cell yield of strain 22A were measured under limited methanol concentrations in the presence of La^3+^ (Fig. 1B). Overall, these parameters were not affected by the presence of La^3+^. With 0.5% methanol, the cell yield was slightly higher in the presence of La^3+^. We generated *mxaF*, *xoxF1*, and *mxaF xoxF1* deletion mutants and examined their growth on methanol. The *xoxF1* mutant and the *mxaF xoxF1* double mutant did not grow on methanol at all, whereas the *mxaF* mutant grew in the presence of La^3+^ (Fig. 1C). We isolated suppressor mutants that regained growth in the absence of lanthanides from the *xoxF* mutant (data not shown). These results indicated that *mxaF* and *xoxF1* encode Ca^2+^-dependent and lanthanide-dependent MDH, respectively, and that *xoxF1* is necessary for *mxaF* expression, as found in strain AM1. Interestingly, the growth yield of the *mxaF* mutant in the presence of La^3+^ was significantly reduced compared to that of the wild type, but its specific growth rate was comparable to that of the wild type (Fig. 1C). The specific growth rate and cell yield of the wild type were not affected by La^3+^ at different concentrations (Fig. 1D). In contrast, those of the *mxaF* mutant increased depending on the La^3+^ concentration up to 30 µM. Thus, its methylotrophic growth was completely dependent on La^3+^. In addition, 30 µM La^3+^ was considered sufficient to achieve the full growth rate depending on XoxF1 in our experimental settings.

### Overview of RNA-seq data.

**FIG 2:**
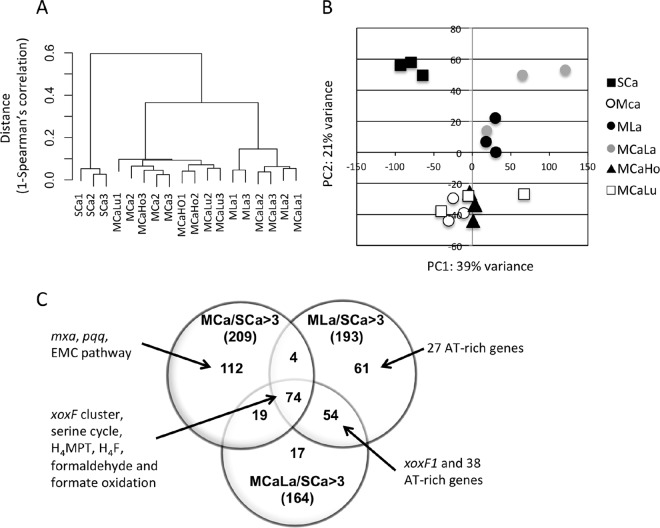
(A) Dendrogram based on the log_2_-transformed expression data of all CDSs, according to Ward’s clustering method as a distance measure with Spearman’s 1 correlation. (B) Principal-component analysis based on the log_2_-transformed expression data of all CDSs. (C) Venn diagram of the number of differentially expressed CDSs. Upregulated CDS numbers under the MCa, MLa, and MCaLa conditions compared to the SCa condition are counted (fold change, >3; *P* < 0.05 under any of the conditions).

We isolated RNA from the cells grown under six conditions, and performed RNA-seq analysis, as detailed in Materials and Methods. As carbon sources, the cells were grown on succinate in the presence of 30 µM Ca^2+^ (SCa condition) or methanol (methanol conditions). For the methanol conditions, metal supplementations with CaCl_2_ (MCa condition), LaCl_3_ (MLa), CaCl_2_ plus LaCl_3_ (MCaLa), CaCl_2_ plus HoCl_3_ (MCaHo), and CaCl_2_ plus LuCl_3_ (MCaLu) were added. The statistics and expression data of the RNA-seq experiment are summarized in Tables S1, S2, and S3 in the supplemental material. Hierarchal clustering (Fig. 2A) and principal-component analysis (Fig. 2B) suggest that the expression profile of the SCa condition was distinct from the methanol conditions. The profiles of the MLa and MCaLa conditions were clearly different from the rest.

10.1128/mSphere.00462-17.7 TABLE S1  Statistics of RNA-seq results, part 1. Download TABLE S1, XLSX file, 0.1 MB. Copyright © 2018 Masuda et al. 2018 Masuda et al. This content is distributed under the terms of the Creative Commons Attribution 4.0 International license.

10.1128/mSphere.00462-17.8 TABLE S2  Statistics of RNA-seq results, part 2. Download TABLE S2, XLSX file, 0.1 MB. Copyright © 2018 Masuda et al. 2018 Masuda et al. This content is distributed under the terms of the Creative Commons Attribution 4.0 International license.

10.1128/mSphere.00462-17.9 TABLE S3  RNA-seq results. Download TABLE S3, TXT file, 2.2 MB. Copyright © 2018 Masuda et al. 2018 Masuda et al. This content is distributed under the terms of the Creative Commons Attribution 4.0 International license.

The fold change analysis for the MCa, MLa, and MCaLa conditions compared to the SCa condition (cutoff, >3-fold changes; *P* < 0.05 under any of the conditions) (Fig. 2C) identified 73 genes that were consistently upregulated under the methanol conditions and were thus considered to be important for methylotrophy, irrespective of the metals (Ca^2+^ or La^3+^). They include those involved in the XoxF cluster (but not *xoxF1*), serine cycle, tetrahydromethanopterin (H_4_MPT) pathway, tetrahydrofolate (H_4_F) pathway, and formaldehyde and formate oxidation. The other distinguishing biological functions were ATPases, chemotaxis proteins, and those involved in flagellar synthesis (Table S3). The upregulated genes under the MCa condition, but not under the MLa and MCaLa conditions, compared to the SCa condition, contained 112 genes involved in the *mxa* cluster, *pqq* cluster, and ethylmalonyl coenzme A (CoA) (EMC) pathway. The other distinguishing functions are ATPases, ABC transporters, and chemotaxis proteins. The genes upregulated under the MLa or MCaLa conditions, but not the MCa condition, included numerous AT-rich genes (GC content of <55%), whereas the average GC content of the strain 22A genome was 69.1%.

### Expression profiles for catabolic methylotrophic pathways.

**FIG 3:**
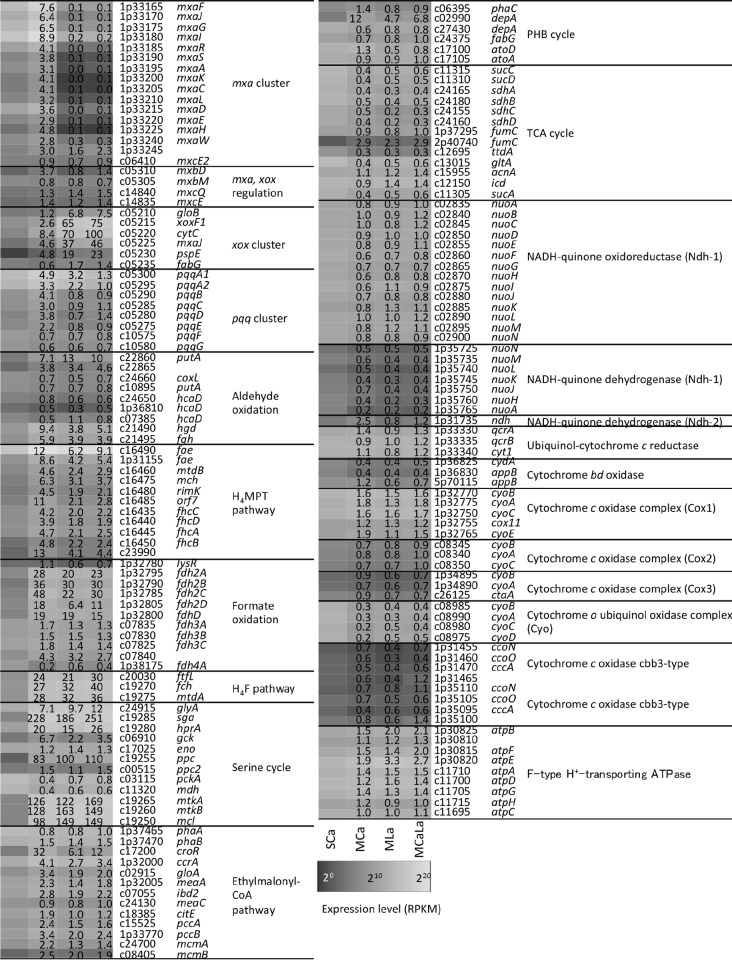
Heat map of the gene expression involved in methylotrophy and related pathways under the SCa, MCa, MLa, and MCaLa conditions. Gene accession numbers are shown without the suffix (Maq22A_). Fold change values against the SCa condition are shown in the heat map.

The expression of the genes related to methylotrophy under the SCa, MCa, MLa, and MCaLa conditions is summarized in Fig. 3. The addition of Ho^3+^ and Lu^3+^ did not cause a significant difference in the expression profile of these genes.

The structure of the *mxa* cluster encoded in the largest plasmid is well conserved compared to that in strain AM1 (see Fig. S1A in the supplemental material). *mxaB* is missing from the cluster, but we found a homologue to AM1 MxaB called MxcE2 with 50% identity in the strain 22A chromosome, which is listed in Fig. 3. Overall, *mxa* genes were highly upregulated under the MCa condition compared to the SCa condition and clearly repressed under the MLa and MCaLa conditions compared to the MCa condition. The expression of *mxcE2* did not change under these conditions.

10.1128/mSphere.00462-17.1 FIG S1  Comparison of structures of the *mxa* and *xox* gene clusters in *M. extorquens* strain AM1 and *M. aquaticum* strain 22A. Download FIG S1, TIF file, 0.1 MB. Copyright © 2018 Masuda et al. 2018 Masuda et al. This content is distributed under the terms of the Creative Commons Attribution 4.0 International license.

The *mxbDM* genes are necessary for *mxaF* expression (23), and the *mxcEQ* genes are necessary for *mxbDM* expression (24) in strain AM1. Both clusters encode sensor kinases (MxcQ and MxbD) and response regulators (MxcE and MxbM). Among these genes, only *mxbD* was upregulated under the MCa condition.

*xoxF1* is clustered with *gloB*, *cytC*, *mxaJ2*, *pspE*, and *fabG* (Fig. S1B). These genes were upregulated under the methanol conditions—particularly strongly in the presence of La^3+^. The two genes upstream of the cluster (*ansB* and c05205) did not respond to methanol. The expression profiles of *mxa* and *xox* clusters showed a striking inverse correlation depending on the presence of La^3+^. The functions of *gloB* and *pspE* associated with *xoxF1* remain unknown and to be characterized. They often cluster with *xoxF4-* and *xoxF5*-type gene clusters (16).

PQQ is synthesized by PqqABCDEFG proteins. The expression of *pqqA_1_A_2_* was more upregulated under the methanol conditions than under the SCa condition. The *pqqBCDE* genes were upregulated only under the MCa condition. The *pqqFG* genes were not responsive to methanol and metals.

Formaldehyde may be oxidized by aldehyde dehydrogenases. We found two genes encoding aldehyde dehydrogenase upregulated under the methanol conditions (*putA* and c22865). Their substrate specificity is unknown. The absence of signal peptides (analyzed by the SignalIP 4.1 server) suggests their intracellular localization; therefore, they are not involved in the direct oxidation of formaldehyde in the periplasm. Dye-linked formaldehyde dehydrogenases were purified from three methylotrophs, and the protein from *M. capsulatus* was identified as sulfide/quinone reductase (MCA2155) or flavin adenine dinucleotide (FAD)-dependent pyridine nucleotide-disulfide oxidoreductase (16). We found three homologues (*hcaD*) with 22 to 30% identities. Their expression was, however, low under the methanol conditions, suggesting their unrelatedness to methylotrophy in strain 22A. In addition, strain 22A has the glutathione-dependent formaldehyde dehydrogenase pathway (glutathione [GSH] pathway); its genome carries *hgd* and *fgh* (see Fig. S2 in the supplemental material) but not the gene encoding glutathione-dependent formaldehyde-activating enzyme (Gfa) (25). Thus, *S*-hydroxymethyl glutathione will be spontaneously produced in strain 22A. The upregulation of these genes under the methanol conditions suggests their involvement in formaldehyde oxidation. The expression of *hgd* was lower under the MLa condition than under the MCa condition. Methylene-H_4_MPT can be formed spontaneously or by the formaldehyde-activating enzyme, which is further transformed to formate via the H_4_MPT pathway. Methylofuran was recently identified in strain AM1, and it carries a C_1_ unit from formyl-H_4_MPT in the pathway (26). The genes involved in the pathway were all upregulated under the MCa condition compared to the SCa condition, but were relatively repressed in the presence of La^3+^ compared to the MCa condition.

10.1128/mSphere.00462-17.2 FIG S2  Predicted pathway for methanol metabolism in *M. aquaticum* strain 22A. The gene names are colored in red (MCa > MLa) and green (MLa > MCa) based on their relative expression. DH, dehydrogenase. Download FIG S2, TIF file, 0.2 MB. Copyright © 2018 Masuda et al. 2018 Masuda et al. This content is distributed under the terms of the Creative Commons Attribution 4.0 International license.

We annotated the molybdenum-dependent formate dehydrogenase complex (*fdh2ABCD*), cytochrome-linked formate dehydrogenase complex (*fdh3ABC* and c07840), and *fdh4A* for formate oxidation. Fdh2 complex was upregulated under the methanol conditions, and the expression of Fdh2C and 2D components was slightly lower under the MLa condition than under the MCa condition. The expression of *fdh4A* was high under the SCa condition and lowest under the MCa condition, the meaning of which is unknown.

The H_4_F pathway converts formate to methylene H_4_F, which is further used as C_1_ supply for the serine cycle. The pathway was upregulated under the methanol conditions; however, expression of the corresponding genes was not responsive to the presence of La^3+^.

### Expression profiles for anabolic methylotrophic pathways.

All genes for the serine cycle (see Fig. S3 in the supplemental material) were upregulated under the methanol conditions, except for *eno* and *ppc2* (Fig. 3). Two molecules of acetyl-CoA produced in the serine cycle are converted to methylmalonyl-CoA through the EMC pathway to supply glyoxylate for the serine cycle (27, 28). Most of the genes in the pathway were upregulated under the methanol conditions, except for *phaA*, *phaB*, and *meaC*. The genes for the EMC pathway were slightly downregulated under the MLa condition compared to the MCa condition. Within the poly-β-hydroxybutyrate (PHB) cycle, *depA* (c02990) was induced higher under the methanol conditions than under the SCa condition. Expression of *depA* (c02990) and *atoD* was higher under the MCa condition than under the MLa condition. The genes for the tricarboxylic acid (TCA) cycle were highly expressed under the SCa condition, which was expected when succinate was the carbon source. Their expression was not responsive to the presence of La^3+^, except for *sdhC* (c24155).

10.1128/mSphere.00462-17.3 FIG S3  Predicted pathway for the serine cycle, TCA cycle, PHB cycle, and ethylmalonyl-CoA pathway in *M. aquaticum* strain 22A. The genes are colored in red when the expression was higher under the MCa condition than under the MLa condition. CA, carboxylase; DH, dehydrogenase. Download FIG S3, TIF file, 0.2 MB. Copyright © 2018 Masuda et al. 2018 Masuda et al. This content is distributed under the terms of the Creative Commons Attribution 4.0 International license.

The expression of one of the NADH dehydrogenase (Ndh-1) clusters encoded in the plasmid pMaq22A-1 (1p35725-1p35765) was relatively low compared to that of the other and was lower under the methanol conditions. The expression of another NADH-quinone dehydrogenase (Ndh-2) was high only under the MCa condition. Ndh-2 does not pump protons, but is important for higher metabolic flux and increased carbon flux into biosynthetic pathways (29, 30). The meaning of this upregulation is currently unclear. Among seven different types of cytochrome oxidases, the expression of components in cytochrome *o*-type ubiquinol oxidase was lower under the methanol conditions than under the SCa condition, and expression was even lower under the MCa condition than under the MLa condition. The expression of F-type ATPase genes was higher under the methanol conditions than under the SCa condition, and some components (*atpB*, *atpE*, and *atpD*) showed slightly higher expression under the MLa and MCaLa conditions than under the MCa condition.

### Lanthanide-dependent expression of MDH-like genes.

**FIG 4:**
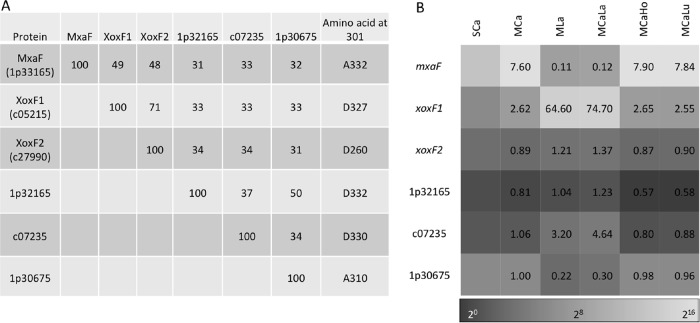
Properties and expression of MDH-like genes found in the strain 22A genome. (A) Pairwise identities of amino acid sequences of MDH-like genes and amino acids corresponding to position 301 (D-) in XoxF from *M. fumarioricum* SolV. (B) Heat map of MDH-like gene expression. Gene accession numbers are shown without the suffix (Maq22A_). Fold change values against the SCa condition are shown in the heat map.

We found four more MDH-like genes besides *mxaF* and *xoxF1* in the strain 22A genome. One of them is named *xoxF2*, the deduced amino acid sequence of whose product has 71% identity to XoxF1 (Fig. 4A). The other three proteins showed 30 to 35% identity to MxaF, XoxF1, and XoxF2. The important amino acid residue (Asp301 in *Methylacidiphilum fumarioricum* SolV XoxF1), which is typical and diagnostic of XoxF-type MDHs and considered necessary for lanthanide ion binding (11), is conserved in four of them.

As already shown in Fig. 3, the expression of *mxaF* and *xoxF1* was clearly switched by the presence of La^3+^ (Fig. 4B), suggesting that strain 22A uses XoxF1 preferentially when La^3+^ is available and uses MxaF only when La^3+^ is absent. The expression of the other four genes was relatively low under these conditions. XoxF2 and 1p32165 showed no clear expression patterns. *xoxF2* might be a pseudogene, because its open reading frame (ORF) seemed to be truncated and does not contain a signal peptide. The expression of c07235 was induced, and 1p30675 was repressed, in the presence of La^3+^. These responses correlate with the presence of amino acids that bind lanthanides: e.g., XoxF1 and c07235 have an Asp residue at position 301 that binds lanthanides, while MxaF and 1p30675 have Ala at position 301 that interacts with calcium.

### Upregulated genes in the presence of lanthanides.

**FIG 5:**
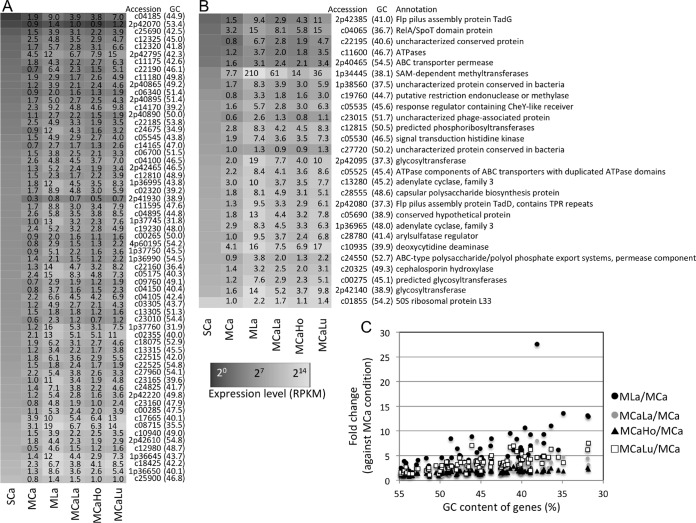
Expression response of AT-rich genes to lanthanides. (A and B) Heat maps of the expression level of the genes with low GC content (<55%) under all conditions. (A) Genes annotated as hypothetical proteins. (B) Annotated genes. The GC content of the gene is shown in parentheses. The genes are sorted to their expression level under the SCa condition. Gene accession numbers are shown without the suffix (Maq22A_). Fold change values against the SCa condition are shown in the heat map. (C) Relationship between gene GC content and fold change in expression against the MCa condition.

We noticed that AT-rich genes showed high relative expression in the presence of lanthanides compared to the MCa condition. Figure 5A and B show the expression of 89 genes with the lowest GC content (<55%) in the strain 22A genome. These genes are scattered in the genome, as shown by their locus tags. The expression of the genes with lower GC content was more impacted by lanthanides, especially under the MLa condition (Fig. 5C). It is known that AT-rich genes are silenced by nucleoid-associated proteins (NAPs) (31, 32). We found 18 NAP genes in the strain 22A genome, and their expression is summarized in Fig. S4 in the supplemental material. None of these genes showed specifically lower expression in the presence of lanthanides compared to the MCa condition, however, suggesting that NAPs may not be involved in the derepression of AT-rich genes.

10.1128/mSphere.00462-17.4 FIG S4  Expression of NAPs presented as a heat map. Gene accession numbers are shown without the suffix (Maq22A_). Download FIG S4, TIF file, 0.1 MB. Copyright © 2018 Masuda et al. 2018 Masuda et al. This content is distributed under the terms of the Creative Commons Attribution 4.0 International license.

**FIG 6:**
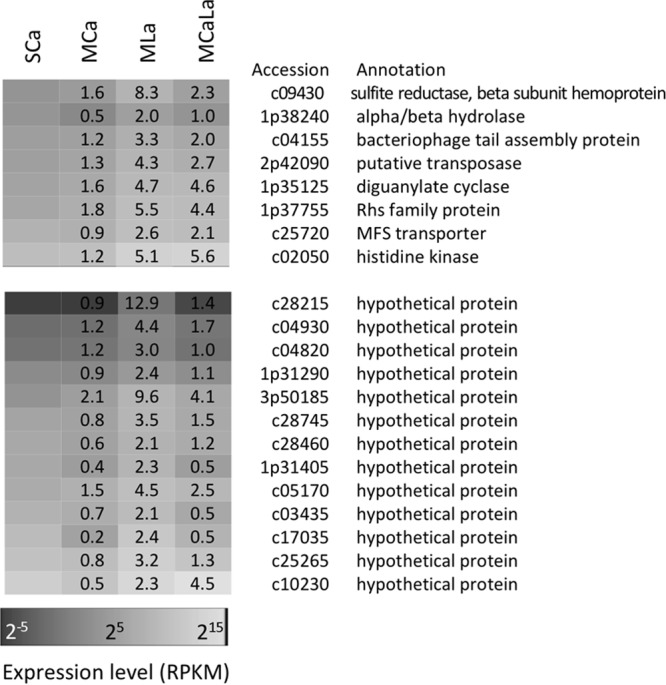
Heat map of expression level of the genes extracted under restrictions of MLa versus MCa >2.5-fold change, MCaLa versus MCa >2.5-fold change, MLa versus SCa >2-fold change, GC content of >60%, and genes not tRNA genes. The genes are sorted to their expression level under the SCa condition. Fold change values against SCa condition are shown in the heat map.

In addition to AT-rich genes, we sought La^3+^-induced genes with the following restrictions: MLa versus MCa, >2.5-fold change; MCaLa versus MCa, >2.5-fold change; MLa versus SCa, >2-fold change; GC content, >60%; genes not tRNA genes. These restrictions extracted 27 genes. They included the aforementioned five genes in the *xoxF* cluster and the quinoprotein alcohol dehydrogenase gene (c07235). The expression of the rest, 16 of which encode hypothetical proteins, is summarized in Fig. 6. The functions of these genes for methylotrophy are currently unknown and remain to be characterized; we describe their general functions in the Discussion section.

### Methanol and formaldehyde oxidation activity in strain 22A.

**FIG 7:**
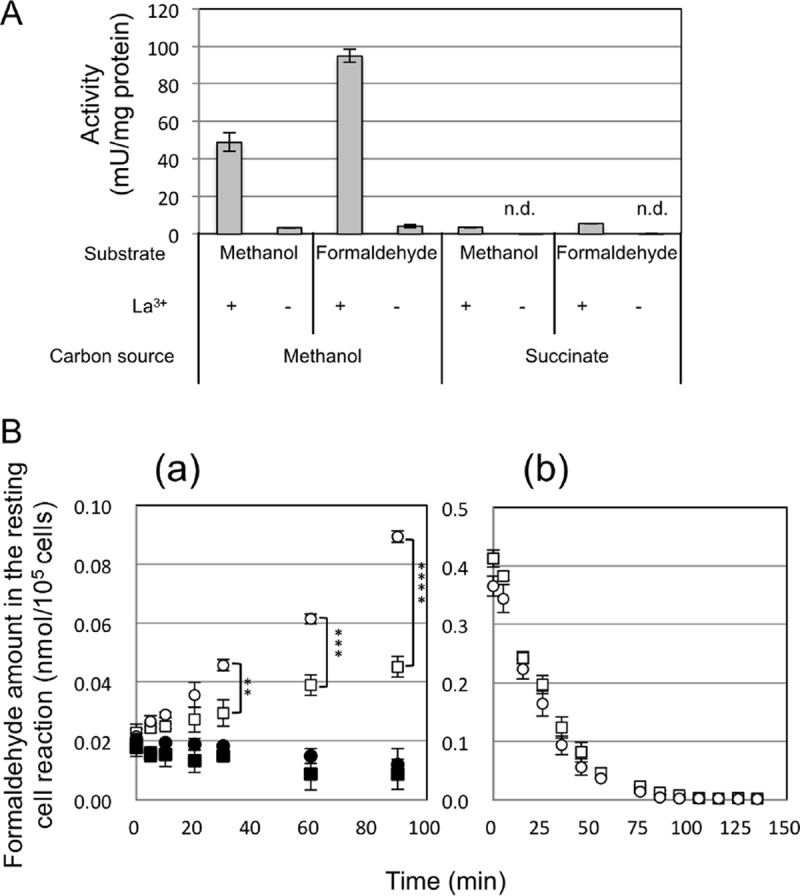
(A) Dehydrogenase activities toward methanol and formaldehyde in the cell-free extracts of strain 22A grown on methanol or succinate, in the presence of 30 µM CaCl_2_, and in the absence and presence of LaCl_3_. Activities were determined from three biological and three technical replicates. n.d., not detected. Error bars show the standard error of the mean (*n =* 3). (B) Formaldehyde production and degradation by resting cells of strain 22A pregrown on methanol in the absence and presence of La^3+^. Production (a) and degradation (b) of formaldehyde by strain 22A cells were measured. Strain 22A was pregrown on methanol in the absence (circles) and presence (squares) of LaCl_3_. Autoclave-killed cells were used as controls (closed symbols). The molar amounts of formaldehyde in the 200-µl reaction mixture in 96-well plates were normalized by total cell numbers (10^5^ cells). Asterisks indicate statistical significance: **, *P* < 0.01; ***, *P* < 0.001; and ****, *P* < 0.0001 (Sidak’s multiple-comparison test).

As shown above, La^3+^ had a particular impact on the expression of MDH genes and formaldehyde oxidation genes. These results prompted us to investigate the difference in the methanol and formaldehyde oxidation activities of cells grown in the presence and absence of La^3+^. The MDH activity in the methanol-grown cells in the presence of La^3+^ was 49 mU·mg^−1^ protein, whereas that in the absence of La^3+^ was 3 mU·mg^−1^ protein (Fig. 7A). The activity toward formaldehyde was also higher in the former (95 mU·mg^−1^ protein compared to 4 mU·mg^−1^ protein). The cells grown on succinate in the presence of La^3+^ showed low activity.

Next, we quantified the formaldehyde accumulation and degradation with resting cells. The formaldehyde accumulation was faster in the cells grown in the presence of Ca^2+^ than in those grown in the presence of La^3+^; the rates were 0.727 and 0.255 pmol formaldehyde·min^−1^·per 10^5^ cells, respectively (Fig. 7B, panel a). The formaldehyde degradation rates were comparable between them; the rates were 7.58 and 7.24 pmol formaldehyde·min^−1^·per 10^5^ cells for cells grown in the presence of Ca^2+^ and La^3+^, respectively (Fig. 7B, panel b).

### PQQ production and biofilm formation.

The relatively low expression of the *pqq* cluster in the presence of La^3+^ (Fig. 3) prompted us to measure PQQ production by strain 22A. As shown in Fig. S5 in the supplemental material, the PQQ concentration in the spent medium was lower in the presence of La^3+^ in strain 22A grown on methanol. Thus, the decreased PQQ production was in line with the lower expression of the *pqq* cluster in the presence of La^3+^; however, its biological meaning is currently unknown since MxaF and XoxF are both PQQ-dependent MDHs.

10.1128/mSphere.00462-17.5 FIG S5  PQQ production by strain 22A grown under different conditions. Strain 22A was grown on either 0.5% methanol, 0.5% succinate, or 0.5% methanol plus 0.5% succinate, in the absence or presence of 30 µM La^3+^ for 7 days. The culture supernatant was subjected to PQQ content measurement by HPLC. Circles, cell yield (wet weight) of 5-ml culture; bars, PQQ concentration of the spent medium. Error bars show SD (*n =* 3). Statistical analysis was carried out by Student’s *t* test only for PQQ production. *P* values are shown only when <0.05. Download FIG S5, TIF file, 0.1 MB. Copyright © 2018 Masuda et al. 2018 Masuda et al. This content is distributed under the terms of the Creative Commons Attribution 4.0 International license.

Related to the upregulation of the diguanylate cyclase gene (Fig. 6), we monitored the colony morphology, motility, and biofilm formation of strain 22A. There was no clear morphological difference in the colonies, no difference in the motility (swimming motility under a microscope, data not shown) of the cells, and no difference in the biofilm formation in the cultures of strain 22A grown on methanol with 30 µM La^3+^ (see Fig. S6 in the supplemental material).

10.1128/mSphere.00462-17.6 FIG S6  Biofilm formation of strain 22A grown on methanol or succinate in the presence or absence of 30 µM La^3+^. Open bars indicate formation in the absence of La^3+^, and gray bars indicate formation in the presence of 30 µM La^3+^. Error bars show SD (*n =* 3). Download FIG S6, TIF file, 0.1 MB. Copyright © 2018 Masuda et al. 2018 Masuda et al. This content is distributed under the terms of the Creative Commons Attribution 4.0 International license.

## DISCUSSION

In this study, we used *M. aquaticum* strain 22A as a model to determine the transcriptomic profile in response to methanol and lanthanides. Strain 22A has some characteristics that differ from strain AM1 in methylotrophy. The strain 22A genome encodes glutathione-dependent formaldehyde dehydrogenase, and the strain does not grow on methylamine due to the absence of methylamine dehydrogenase. In addition, strain 22A grows on glucose but strain AM1 does not. Despite these differences, the MDH systems of MxaF and XoxF1 (and other MDH-like genes as well) are commonly found in *Methylobacterium* genomes, and almost all of the components of the metabolic pathways are shared in common.

Ho^3+^ and Lu^3+^ did not impact the growth rate of strain 22A and the expression of methylotrophy genes compared to the MCa condition (Fig. 1A; Table S3). Since *xoxF1* in strain AM1 is induced only by light lanthanides (13), these heavier lanthanides may not be recognized by strain 22A and its XoxF1 protein as well, probably due to their different ion radii. The strain 22A *mxaF* mutant showed comparable specific growth rates due to intact *xoxF1* in the presence of La^3+^, but its growth yield was significantly lower than that of the wild type (Fig. 1C). This is discussed below with the expression data and enzyme activities. The strain 22A mutants of *xoxF1* and *xoxF1 mxaF* could not grow on methanol at all (Fig. 1C), and *xoxF2* might be a pseudogene. Thus, the necessity of *xoxF1* for *mxaF* expression seemed to also occur in strain 22A as in strain AM1, and only *mxaF* and *xoxF1* are able to support growth on methanol in strain 22A, whereas a strain AM1 *mxaF xoxF1 xoxF2* triple mutant can still grow on methanol in the presence of La^3+^ (13, 33). The strain 22A *mxaF* mutant required more than 30 µM La^3+^ to achieve its full growth rate (Fig. 1D), whereas 1 µM La^3+^ was enough for an AM1 *mxaF* mutant (33). These are the distinct differences in mutant phenotypes in strains 22A and AM1.

La^3+^ had a significant impact on the expression of methylotrophy genes in strain 22A. The most striking contrast in gene expression was observed for the switching of the *mxa* and *xox* clusters (Fig. 3). Specifically, *mxaF* and *xoxF1* showed approximately 71-fold and 24-fold induction, depending on the presence or absence of La^3+^, respectively (Fig. 4). As suggested in strain AM1 (6, 13), *mxbD*, which has higher expression under the MCa condition than under the MLa condition in strain 22A, is considered to regulate the switching. The ligand for MxbD is currently unknown. The expression of *mxcE2* did not change under these conditions, although the *mxaB* homologue in *Methylomicrobium buryatense* strain 5GB1C was reported to be lanthanide responsive and partially involved in MDH switching (14).

In addition to the MDH systems, the formaldehyde oxidation systems of GSH and H_4_MPT pathways were downregulated in the presence of La^3+^ compared to the MCa condition. This might be explained by the direct oxidation of formaldehyde by XoxF (11, 16). The high activity toward formaldehyde (Fig. 7A) and the reduced formaldehyde production in cells grown in the presence of La^3+^ (Fig. 7B) support this explanation. When XoxF oxidized formaldehyde in the presence of La^3+^, it would produce more reduced cytochrome *c* than MxaF produces under the MCa condition. The decreased formaldehyde generation by MDHs may lead to decreased induction of the H_4_MPT and GSH pathways, which further leads to the decreased generation of NAD(P)H. These differences might cause the decreased expression of the second NADH dehydrogenase (Fig. 3). It was reported that strain AM1 cells contain more ATP when grown on methanol and more reducing equivalents [NAD(P)H] when grown on succinate (34). Thus, the higher expression of the ATPase complex under the methanol conditions (Fig. 3) was in accordance with the results from strain AM1, and the slight increase under the MLa condition suggested even higher ATP levels under this condition. These changes in the formaldehyde oxidation and respiratory chain could be a response to the direct oxidation of methanol by XoxF1 and the concomitant decreased production of NAD(P)H.

Even when the concentration of methanol is limited, strain 22A showed comparable growth rates and cell yield in the presence and absence of La^3+^ (Fig. 1B). This result suggested that something other than methanol is the limiting factor for the growth rates. Since the direct oxidation of methanol to formate by XoxF1 allows less energy to be conserved [compared to the sequential oxidation of methanol by the MxaF and NAD(P)-dependent formaldehyde dehydrogenation pathways], one may expect a higher growth rate and lower cell yield in the presence of La^3+^, but this was not the case under our experimental condition. The lower cell yield of the *mxaF* mutant compared to the wild type in the presence of La^3+^ (Fig. 1C) might suggest that *mxaF* also takes part in the methanol metabolism in the wild type, even in the presence of La^3+^, or that lower energy conservation decreased the cell yield. Interestingly, this lowered cell yield does not occur in strain AM1 (10, 13). The MDH activity detected in strain 22A cells grown on methanol in the absence of La^3+^ (3 mU·mg^−1^ protein) was considerably lower than that in the presence of La^3+^ (49 mU·mg^−1^ protein) (Fig. 7A). These activities reflect the activity of MxaFI and XoxF1 (Fig. 4), but are contradictory to the comparative growth rates (Fig. 1C) of the wild type. The cell extract might contain unknown inhibitory factor(s) for MxaFI, or the enzyme might be unstable. It is also known that the range of MDH activities based on the phenazine methosulfate (PMS) and dichlorophenol indophenol (DCPIP) assay system is variable depending on the protocols (64 to 540 mU·mg^−1^ protein) (35). Thus, biochemical and enzymological characterization of MDHs in strain 22A is necessary, and cellular metabolic flux analysis is required to determine the carbon flow in the methanol metabolism in the presence of lanthanides.

In addition to MDH genes, other putative PQQ-containing dehydrogenase genes (c07235 and 1p30675) were also responsive to La^3+^ (Fig. 6). In strain AM1, ExaA (*META1_1139*) was revealed to be a lanthanide-dependent ethanol dehydrogenase that can oxidize ethanol and acetaldehyde, as well as methanol and formaldehyde, which conferred the growth of an *mxaF xoxF1 xoxF2* mutant on methanol (33). The protein is most homologous to c07235 in strain 22A (37% identity). The substrate specificity and biological function of the proteins encoded by these genes are currently unclear.

The presence of lanthanides induced expression of AT-rich genes in strain 22A, whose functions are unlikely to be important for methylotrophy (Fig. 5); this is intriguing as a biological response to lanthanides, however. Since AT-rich regions in bacterial genomes could be xenogeneic, controlling their expression is pivotal, as seen in *Pseudomonas aeruginosa* (36) and *Escherichia coli* (37). In *E. coli*, the abundance of NAPs depends on the growth phase, and the abundance of H-NS, HU, and Lrp sharply peaks in the exponential growth phase (38). Thus, we could not rule out that the small differences in the growth phase of the harvested cultures might affect the abundance of the transcripts of these genes. As their expression levels were not specifically low under lanthanide-containing conditions (Fig. S4), however, NAPs may not be involved in AT-rich gene induction. Another hypothesis is La^3+^-induced DNA structural modification and the resultant enhanced transcription. Double-strand DNA forms two conformations: right-handed B-DNA (the natural form *in vivo*) and left-handed Z-DNA. Although Z-DNA formation requires extreme ionic strength (4 M NaCl), it is correlated with transcriptional activity. Recently, it was shown that lanthanide ions at millimolar levels were loaded to the grooves of DNA and stabilize the Z-DNA conformation with sequence sensitivity (39). It is unknown whether this is also the case for the observed phenomenon.

The strain 22A genome contains 28 genes for diguanylate cyclase or phosphodiesterase, among which 1p35125 showed the highest expression under the MLa condition (Fig. 6). This protein contains the GGDEF domain, regulating cell surface structures, including exopolysaccharide synthesis, biofilm formation, and motility (40). It is also encoded near *xoxF4*- and *xoxF5*-type genes in some methylotrophic bacteria (16), although this is not the case for strain 22A. Lanthanides induced exopolysaccharide production in *Bradyrhizobium* sp. strain MAFF211645 (41). We did not recognize any difference in the colony morphology, motility, and biofilm formation (Fig. S6) in strain 22A. Thus, the function of the gene remains unclear at the moment.

The *rhs* (rearrangement hot spot) gene 1p37755 is located in a cluster containing two other *rhs* genes. These three *rhs* genes flank two AT-rich regions containing 1p37745, 1p37750, and 1p37760 with high expression in the presence of lanthanides (Fig. 5). Thus, the expression of *rhs* might be influenced by the upregulation of these AT-rich genes. Homologues of most, if not all, of these genes (*rhs* and AT-rich genes) are conserved even in different classes of bacteria. Furthermore, these *rhs* genes are linked to type VI secretion systems (T6SS), suggesting a possible mechanism of Rhs delivery (42, 43). A gene encoding the bacteriophage tail assembly protein involved in T6SS was also found to respond to La^3+^ (c04155, Fig. 6). Thus, there might be a link between lanthanides and T6SS induction, and it is tempting to hypothesize that lanthanides induce the competition machinery in *Methylobacterium*.

The amino acid sequence of c02050, annotated as “histidine kinase,” contains a signal peptide and an EF-hand calcium-binding motif, but no kinase domain. EF-hand motifs are known to bind lanthanides (44, 45). The gene is conserved in many *Methylobacterium* and *Bradyrhizobium* genomes with high homology (>55% identity) and is also annotated as coding for a “calcium-binding protein.” Its La^3+^-dependent induction and possible metal binding imply that it binds lanthanides in the periplasm. The function of the gene is under investigation.

In conclusion, the presence of La^3+^ impacted the expression of both MDH genes and genes involved in the downstream pathway, possibly due to formaldehyde oxidation by XoxF1. In addition to methylotrophy genes, AT-rich genes and those possibly involved in cell survival were found to be upregulated in the presence of lanthanides. The validation of the importance of these genes through biochemical and genetic characterization will enhance our understanding of microbial methylotrophy in environments where lanthanides are present.

## MATERIALS AND METHODS

### Generation of MDH gene deletion mutants of *M. aquaticum* strain 22A.

Strain 22A *mxaF* and *xoxF1* were subjected to deletion mutagenesis. Genomic regions containing each of these genes (ca. 4 kb) were PCR amplified using primers mxaf-UP (5′-TCGAGCTCGGTACCCGAGGTCTCGACCGGCATCGCCTCGGGGAA-3′) and mxaf-DOWN (5′-CTCTAGAGGATCCCCTCGACTGGTCGAACCGCATCGCGACCTT-3′) for *mxaF* and xoxf-UP (5′-TCGAGCTCGGTACCCACCCATGTCCACCCCGACCACGTGC-3′) and xoxf-DOWN (5′-CTCTAGAGGATCCCCAGCTTCACCTTCAGCTCGTCGGCGA-3′) for *xoxF1* and cloned into the SmaI site in pK18mobSacB (46) using the In-Fusion cloning kit (TaKaRa Bio Co.). Next, the regions of the ORFs were eliminated by inverse PCR with KOD DNA polymerase (Toyobo Co.) using primers Inv-mxaf-UP (5′-GTTCAGCGTCTCCAATCGGC-3′) and Inv-mxaf-DOWN (5′-TCCGCCGATCCAGCGCTCCT-3′) for *mxaF* and Inv-xoxf1-UP (5′-GGATTCCTCCGACAGGTGCA-3′) and Inv-xoxf1-DOWN (5′-CGCGCGCCCGCACATCAGAC-3′) for *xoxF1*, and the amplified fragments were circularized with polynucleotide kinase and T4 DNA ligase. The transformation of the plasmids into strain 22A, selection of double-crossover mutants, and diagnosis were carried out as described previously (47).

### Cultivation conditions for RNA-seq.

*M. aquaticum* strain 22A was grown in 100 ml mineral medium (MM; prepared without CaCl_2_) (47) containing 0.5% (wt/vol) succinate and 30 µM CaCl_2_ (called the SCa condition) or 0.5% (vol/vol) methanol (the methanol condition) with different metal supplementations in 500-ml glass culture flasks. As metal supplements, 30 µM each CaCl_2_ (the MCa condition), LaCl_3_ (MLa), CaCl_2_ plus LaCl_3_ (MCaLa), CaCl_2_ plus HoCl_3_ (MCaHo), and CaCl_2_ plus LuCl_3_ (MCaLu) was added. The flasks were washed with 1 M HCl prior to use. For the MLa condition, we did not add CaCl_2_, but the medium was not free from Ca^2+^ since it is impossible to eliminate calcium ions completely; therefore, the cells grew normally even under the MLa condition. The flasks were shaken at 200 rpm at 28°C for 38 to 84 h. In the log phase of growth (optical density at 600 nm [OD_600_] of 0.3 to 0.4), the cells were harvested by centrifugation at 6,500 × *g* at 4°C for 5 min and frozen with liquid nitrogen. The cultivation was carried out in triplicate.

For growth experiments, strain 22A and its MDH gene deletion mutants were grown in 200 µl MM containing 30 µM CaCl_2_ prepared in 96-well plates at 28°C with rotary shaking at 300 rpm. Growth (OD_600_) was measured using a microplate reader (PowerScan HT, DS Pharma Biomedical) without dilution and a factor of 3.42 to convert the readings to a 1-cm light path. The readings of the microplate reader were linear up to an OD_600_ of 1.0 (3.42 with a 1-cm light path).

### RNA-seq.

The frozen cells were subjected to total RNA extraction by the hot phenol extraction method (48). DNA was digested with RQ1 RNase-free DNase I (Promega, Fitchburg, WI). Next, rRNA was depleted using Ribo-Zero rRNA removal kits (Gram-negative bacteria) (Epicentre) according to the manufacturer’s protocol. The rRNA-depleted samples were subjected to single-read sequencing on an Illumina HiSeq 2500 system.

### Mapping and data analysis.

The reads were mapped to the sequences of six replicons of the strain 22A genome and the coding DNA sequence (CDS) list containing rRNA sequences using BWA (49) at Maser (https://cell-innovation.nig.ac.jp). The reads mapped to ribosomal RNAs were subtracted from the total mapped reads to count reads mapped to CDSs and to calculate reads per kilobase per million (RPKM) based on read counts to CDSs (but not total reads). Since samples of MLa and MCaLu were sequenced on a 100-base platform, the read data were trimmed to 36 bases for mapping. Differential abundance analysis was done with DESeq2 1.18.0 (50) of the R package (version 3.3.3) (51).

### MDH activity assay.

Strain 22A was cultured on 0.5% (vol/vol) methanol or 0.5 (wt/vol) succinate in 1 liter MM in the absence or presence of 30 µM LaCl_3_ at 28°C for 3 days. The cells were collected, suspended in 50 mM morpholineethanesulfonic acid (MES)–NaOH buffer (pH 5.5), and disrupted with a Mini-BeadBeater (BioSpec 3110BX; Ieda Trading Corporation). The samples were centrifuged at 10,000 × *g* at 4°C for 10 min. The supernatant was used as cell extract and subjected to the MDH activity assay. The reaction mixture in 96-well plates contained 158 µl of 100 mM Tris-HCl (pH 9.0), 2 µl of 1.5 M ammonium chloride, 10 µl of 6.6 mM phenazine methosulfate (PMS), 10 µl of 1 mM dichlorophenol indophenol (DCPIP), and 10 µl of enzyme solution. After incubation at 30°C for 5 to 10 min when the dye bleaching stabilized, 10 µl of 20 mM methanol was added to initiate the reaction. The change at 600 nm was monitored using a microplate reader (PowerScan HT). Protein concentrations were measured by the Bradford method with bovine serum albumin as the standard. The specific activity was calculated based on a molar extinction coefficient at 600 nm for DCPIP of 19,000 M^−1^·cm^−1^ and a factor of 1.62 to convert the microplate readings to a 1-cm light path. One unit of activity was defined as the enzyme amount that catalyzes the oxidation of 1 µmol of the substrate. Instead of methanol, the same concentration of formaldehyde (prepared by autoclaving paraformaldehyde solution) was also tested as a substrate.

### Formaldehyde production and degradation in resting cell reaction.

Strain 22A cells, grown on solid MM containing 0.5% methanol in the presence and absence of 30 µM LaCl_3_ at 28°C for 2 days, were washed with HEPES buffer (10 mM, pH 7.0). The cell density was adjusted to an OD_600_ of 0.1 (formaldehyde production) or 0.5 (formaldehyde degradation) in HEPES buffer, and the cells grown in the presence of LaCl_3_ were suspended in the buffer containing 30 µM LaCl_3_. The cell suspensions were aliquoted to 96-well plates (180 µl). For formaldehyde production, 20 µl of 20% methanol was added, and the plates were incubated at 28°C for 90 min. At the appropriate time, 20 µl of trichloroacetic acid was added to terminate the reaction. The plates were centrifuged at 2,000 × *g* for 5 min, and 100 µl of the supernatant was transferred into new plates containing 100 µl of 15% (wt/vol) ammonium acetate, 0.3% (vol/vol) acetic acid, and 0.2% (vol/vol) acetylacetone (52). The plates were incubated at 30°C for 30 min, and absorbance at 410 nm was measured using a microplate reader (PowerScan HT). A solution of formaldehyde (0 to 1 mM) served as the standard. For formaldehyde degradation, 20 µl of 5 mM formaldehyde was added to the wells, and the subsequent procedure was performed the same as described above. The cell suspensions were spread onto R2A plate medium for CFU determination. As controls, autoclave-killed cell suspensions (120°C for 15 min) were used. The data were analyzed by Sidak’s multiple-comparison test using Prism 6.0f (GraphPad Software, Inc.).

### Analytical methods.

PQQ content in the spent culture supernatant of strain 22A grown under different conditions was measured by high-performance liquid chromatography (HPLC) on a chromatograph equipped with a fluorescence detector as reported previously (53). For biofilm quantification, strain 22A was grown on 0.5% (vol/vol) methanol or 0.5% (wt/vol) succinate in the absence or presence of 30 µM LaCl_3_ in 200 µl MM prepared in 96-well plates at 28°C for 1 week. On days 3 and 7 after inoculation, the media were removed and the wells were washed with water three times. Next, the plates were dried for 45 min at room temperature, and 200 µl of 0.5% crystal violet was added. After 45 min of incubation at room temperature, the wells were washed five times, and 200 µl of 95% ethanol was added. The absorbance of the samples at 595 nm was measured, and *A*_595_ (OD_600_ of the culture) was used for evaluation of biofilm formation. Statistical analysis was done with Prism 6.0h (GraphPad Software, Inc.).

### Data availability.

The RNA-seq raw data have been deposited into DDBJ under GenBank accession no. DRA002908.
