# Supplementary figures and images for: Lanthanide-Dependent Regulation of Methylotrophy in Methylobacterium aquaticum Strain 22A
Source: mSphere. 2018 Jan 24;3(1):e00462-17. doi: 10.1128/mSphere.00462-17 (PMC5784242; doi:10.1128/mSphere.00462-17)

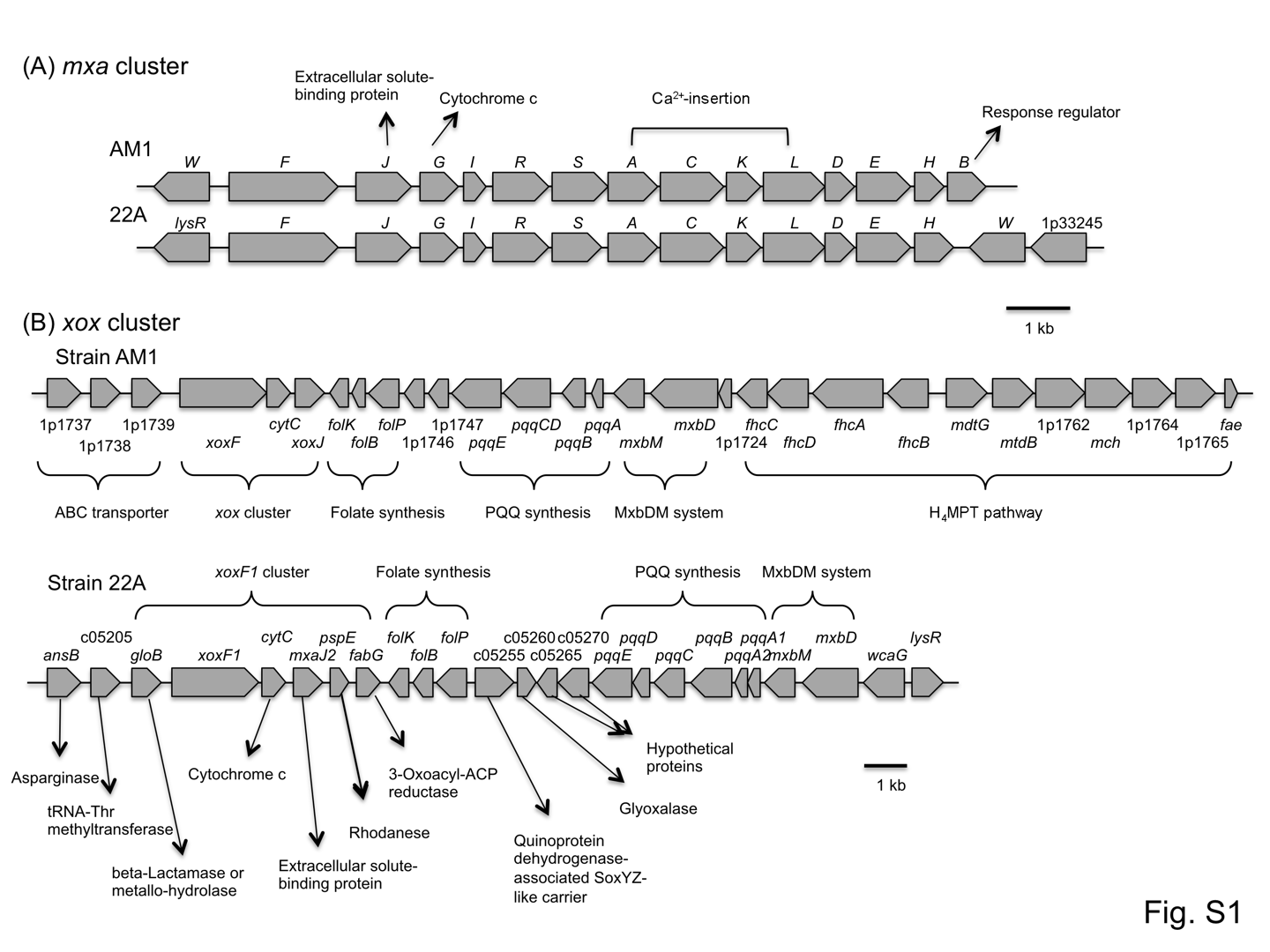

Supplement: FIG S1 [file sph001182462sf1.tif]

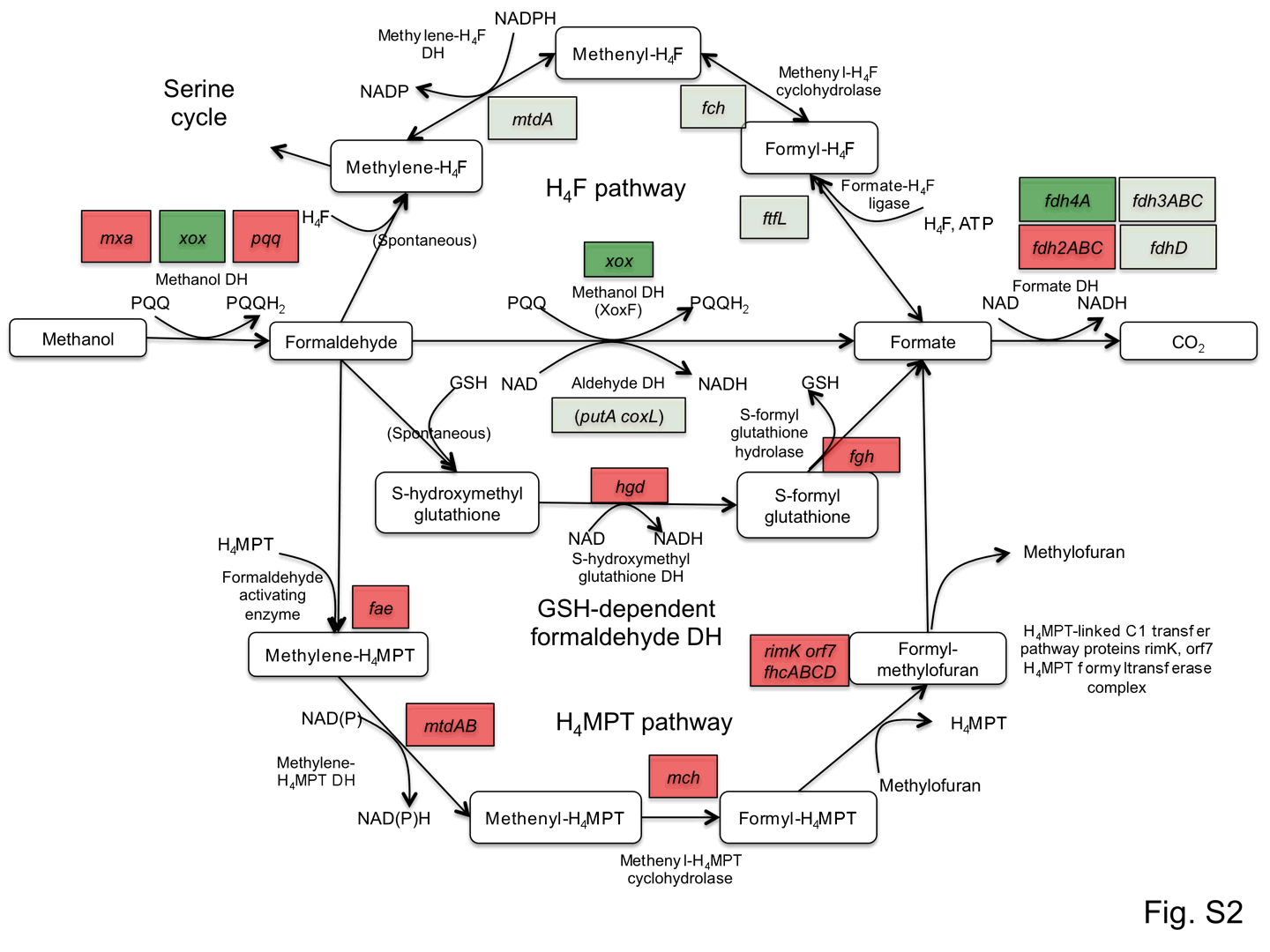

Supplement: FIG S2 [file sph001182462sf2.tif]

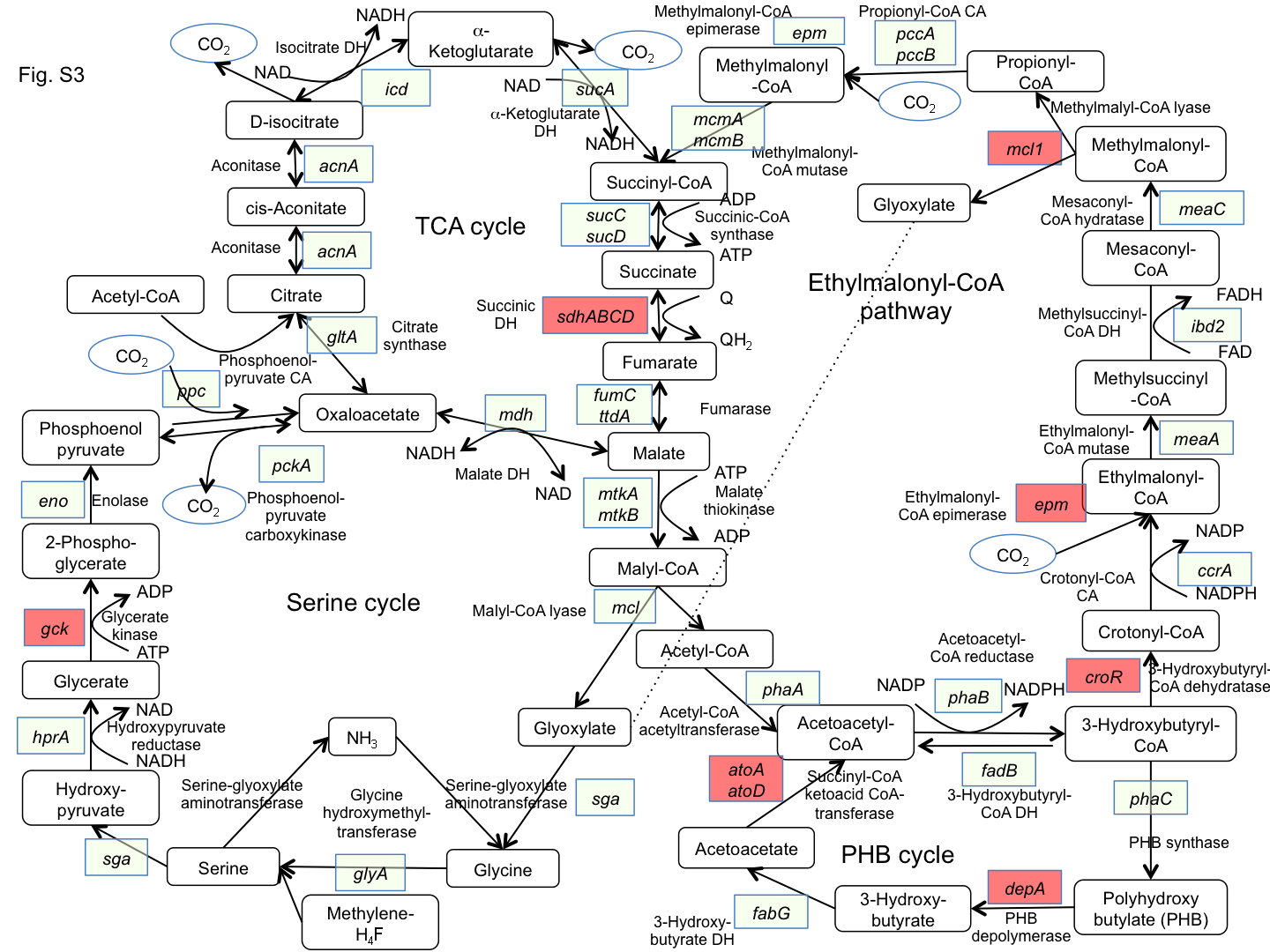

Supplement: FIG S3 [file sph001182462sf3.tif]

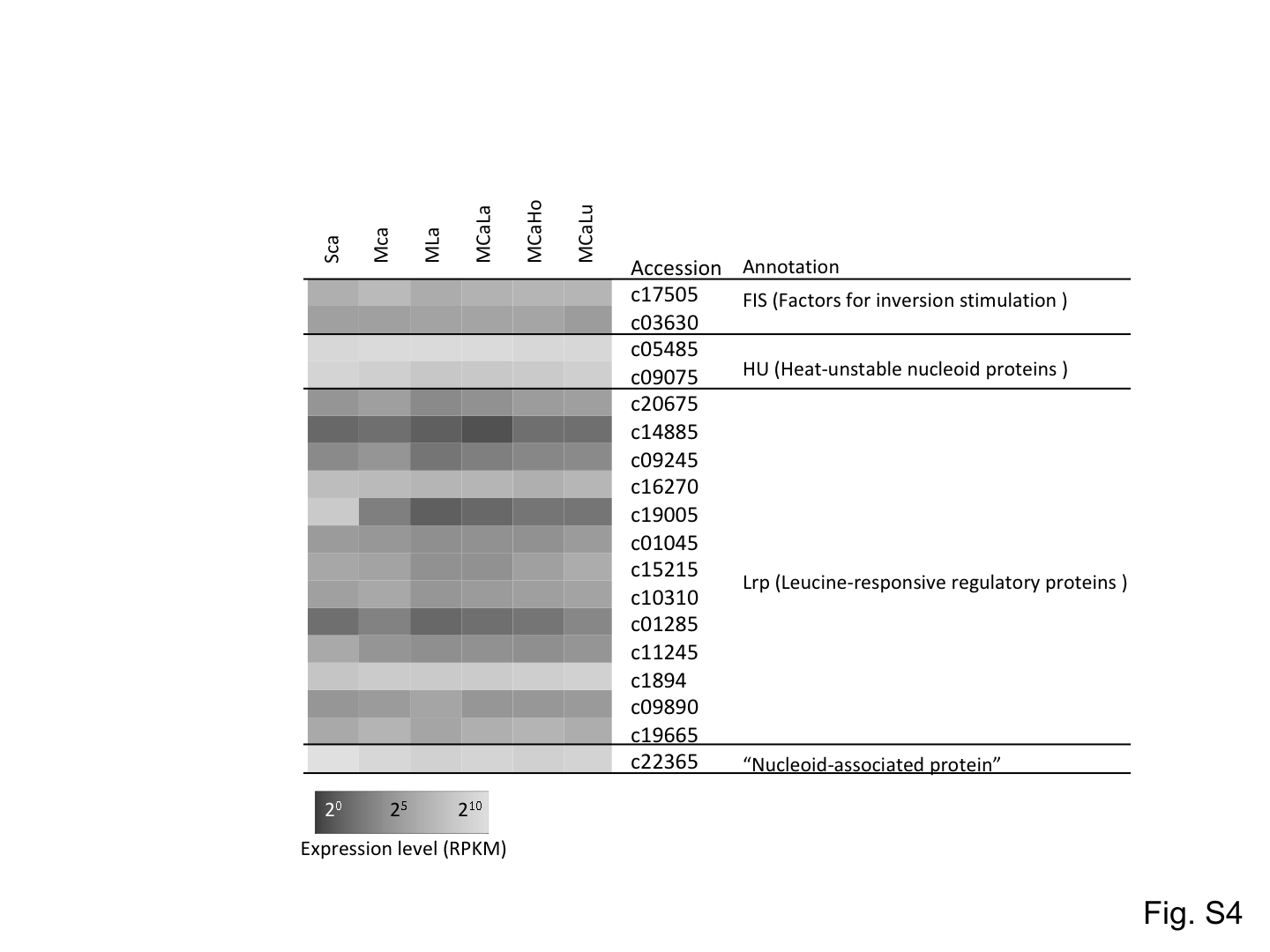

Supplement: FIG S4 [file sph001182462sf4.tif]

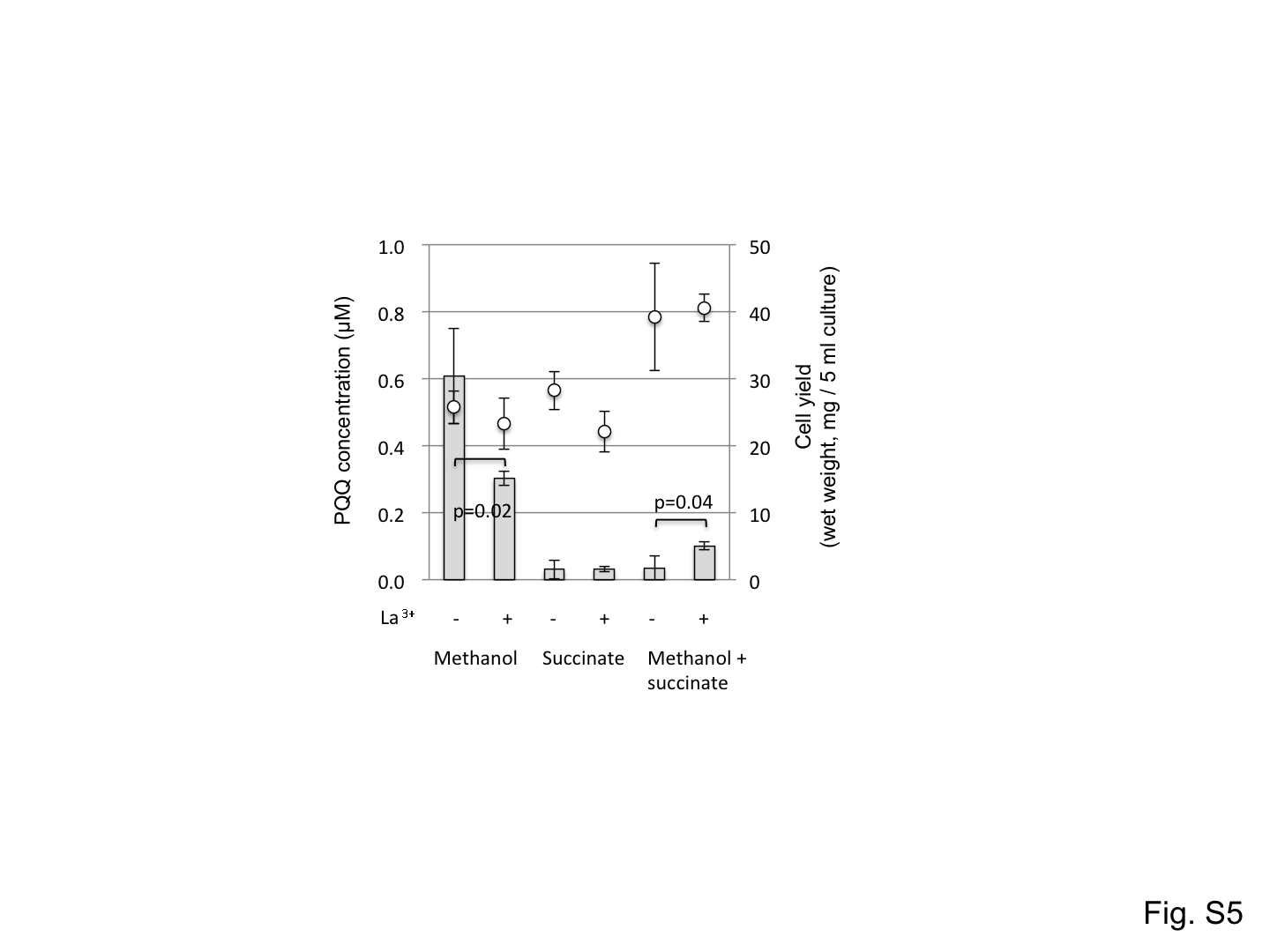

Supplement: FIG S5 [file sph001182462sf5.tif]

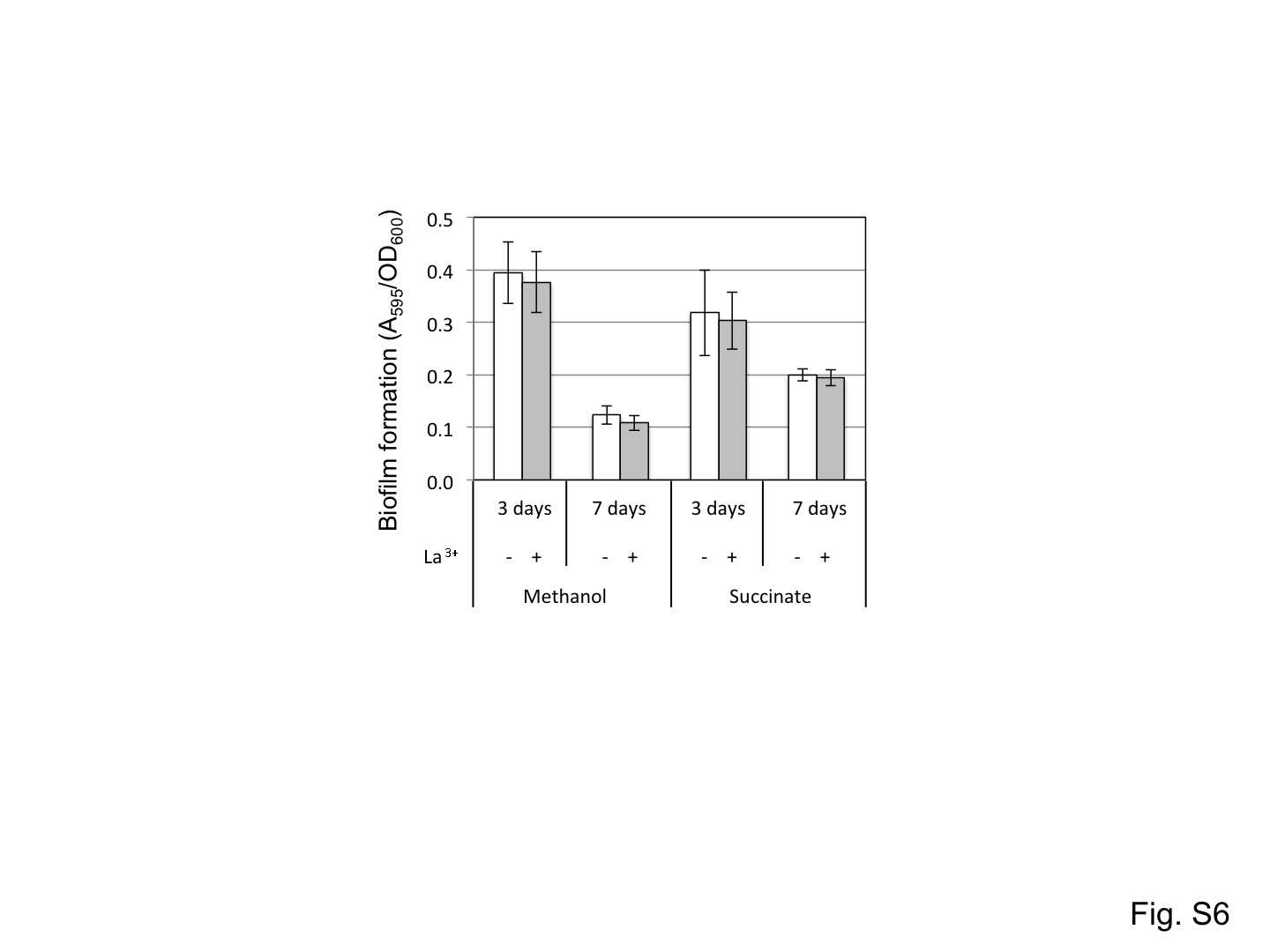

Supplement: FIG S6 [file sph001182462sf6.tif]
